# Mechanisms of Social Interaction and Virtual Connections as Strong Predictors of Wellbeing of Older Adults

**DOI:** 10.3390/healthcare10030553

**Published:** 2022-03-16

**Authors:** Keya Sen, Victor Prybutok, Gayle Prybutok, William Senn

**Affiliations:** 1 School of Health Administration, College of Health Professions, Texas State University, San Marcos, TX 78666, USA; 2 Toulouse Graduate School, University of North Texas, Denton, TX 76203, USA; Victor.Prybutok@unt.edu; 3 Department of Rehabilitation and Health Services, College of Health and Public Service, University of North Texas, Denton, TX 76203, USA; gayle.prybutok@unt.edu; 4 Tarleton State University, Stephenville, TX 76402, USA; wsenn@tarleton.edu

**Keywords:** older adults, quality of life, social support, community engagement, depression

## Abstract

Socially engaged older adults are less likely to decline in health and happiness and have a higher quality of life. Building upon this premise, examination was conducted on the domains of social determinants of health, specifically the social and community context per Healthy People 2030 objectives. These mechanisms of social interaction, in the form of group activities, community engagement, and virtual interactions via email or text message, were assessed using hierarchical regression analysis to find out their association with wellbeing, depression symptoms, and cognition of older adults. The data included a total of 4623 sample of older adults from the National Health and Aging Trend Study (NHATS) Round 8. The results showed that social support explained a 40.3% unique variance on wellbeing. The use of text message and email had a moderating effect on community engagement and self-reported depression level in older adults. Findings suggest that community programs, shared group activities, or technology training workshops can improve social interaction and support cognition and reduce depression in older adults. Directions for future research include examining human behaviors and perceptions and increasing technology training sessions to promote independence of older adults and increase their social connections. In addition, participant involvement in interventions would enhance the possibility of success of such endeavors.

## 1. Introduction

Purposeful activities for social interaction, community engagement, and the use of technology via the Internet can foster wellbeing and connect the older adults to broader social networks and to the rest of the society [1]. With the increased number of older adults in the United States living alone [2], the deleterious effects of social isolation and depression have been exacerbated [3], conflicting with the quality of life. Social isolation contributes to psychological distress by reducing sources of support, such as family and friends, thereby increasing the need for personal resources, such as self-efficacy and engagement in health behaviors [4]. Social disconnectedness of the elderly, in the absence of physical contact or loss of partners in death, severely shrinks their social network [5], thus increasing the need to promote social and community support and build up resilience through age-friendly programs based on available community resources [6].

Wellbeing implies good health with a sense of happiness and trust [7] that can be fostered with social support [1]. The domains of Social Determinants of Health (SDOH) per the Healthy People 2030 objectives “the social and community context” emphasizes the idea of social interaction [7]. This implies a physical or virtual connection with friends, neighbors, or family, which can be further enhanced through group activities, community engagement, or the use of technology for email or text messages. The National Health and Aging Trend Study (NHATS) dataset (https://www.nhats.org) helped in the conceptual development of a model that is driven by a psychosocial theory on aging, namely Activity Theory [7], in our effort to create a balance of the unlimited number of independent constructs based on a fixed set of questions in the NHATS survey. The NHATS online dashboards and companion chartbook track trends were used to analyze the variables as is described in the NHATS codebook to understand the social mechanisms and late-life trajectory. The structure of these indicators matches the proposed indicators of the Healthy People 2030 social domain health objectives based on mental health and a healthier social environment to optimize the quality of life. We think that social support can redress social isolation, promoting a heightened feeling of self-realization and life satisfaction, fostering psychological and physiological health [2]. Hence, it is likely that wellbeing can sustain cognitive ability and reduce depressive symptoms among older adults relative to those with less social support [6].

From the geriatric standpoint, the quality of life is impaired when wellbeing is affected by the onset of cognitive dysfunction, comorbidities, and disability [8] This affects the performance of social roles, which makes the reciprocity of relationships, social activity participation, mealtime enjoyment, and the perceived friendliness of formal and informal caregivers [4] as important components of wellbeing to their lives. Reduced social engagement or higher levels of negativity across the social network is often associated with lower life satisfaction and chronic allostatic load that might stimulate neuroendocrine dysregulation, causing long-term problems related to stress response and hypertension [9].

### 1.1. Study Purpose

This study was conducted to investigate the social determinates of health and their association with wellbeing in older adults. Specifically, group activities and community engagement has been shown to influence wellbeing. The use of technology, especially in the form of emails and text messages, was examined to see how it impacts memory and reduces depression in older adults.

### 1.2. Background

People with multifaceted social connections are inclined towards inclusive behaviors under peer influence [10]. The motivation to engage in work and be active with others fosters a healthy lifestyle that reduces the risk of the occurrence of depressive symptoms and additive behaviors, such as smoking or drinking [11]. High-quality relationships decrease the propensity to dementia [12] and suicidal ideation [13], also adding to physical activity. Hence, older adults who are socially connected even when living alone have a higher life satisfaction, cognitive stability, and reduced depression [14].

### 1.3. Facets of Social Support

#### 1.3.1. Participatory Activities and Related Perceptions

Organized group activities are effective forms of social support, promoting wellbeing available as a paid service or on a voluntary basis either in senior centers or exercise clubs, in family or friendly gatherings, or religious or recreational centers [15]. However, these activities are less effective for people with a neurotic or anxious personality who have unrealistic expectation about relationships [16]. The social attachment framework is subjective, as the question remains as to whether older adults are different from others in their social networks, [17] as they continue to remain embedded in traditional neighborhood communities away from vibrant social media most of the time [18]. 

#### 1.3.2. Technology Use

Not all older adults use digital media to keep in touch with traditional family groups and neighbors even if they are not networked individuals [19]. Unlike young adults, they are less adept at leveraging the advantages of communication technology to connect more effectively and overcome the barriers of time and space to discover new life experiences [20] This could be due to lack of technology skills, economic constraints, or lack of community initiatives. The development of a user-friendly interface through an affordable communication network can virtually connect the older adults [21] to their social world.

#### 1.3.3. Cognition

Older adults involved in online social networking via social media, emails, and text messages have better cognition, including memory and fine motor skills, such as hand–eye coordination [22]. Even among dementia patients, the use of e-health technologies [23] and interventions are interest-generating activities [24], which supports the fact that computer training programs could improve the cognitive performance of older adults and activate their social connection with computer professionals and peers, thereby fostering well-being [25].

#### 1.3.4. Community Engagement

Shared territories improve human interactions [26], contributing to neighborhood networks that support community cohesion and individual wellbeing [27]. Such social spaces connect older adults to everyday neighborhood activities and interactions [28]. Moving to a retirement community often involves a major life transition for older adults that requires psychosocial adjustment to get involved with fellow residents [29]. This transition, demanding new social behaviors and cultural adaptation, is easy when staff in the facility are ready to cooperate and support the new residents in an age-friendly manner [30]. Hence, community engagement provides an enhanced opportunity to inclusion, embracing diversity in a multicultural setting.

#### 1.3.5. Mobility

Travel behavior and mobility are positively associated with life satisfaction [31]. Older adult emotions are positively affected by mobility factors, such as vehicle availability or being able to drive or walk to seniors’ centers and meet with friends. Lower levels of mobility due to physical impairments, sedentary lifestyle, and long hours of TV viewing as well as assistance with activities of daily living (ADLs) are positively associated with heightened depressive symptoms [32]. As mobility relates to motor control and maintenance of brain volume, being able drive or workout with friends promotes functional independence [33].

### 1.4. Research Question and Hypothesis

**Figure 1:**
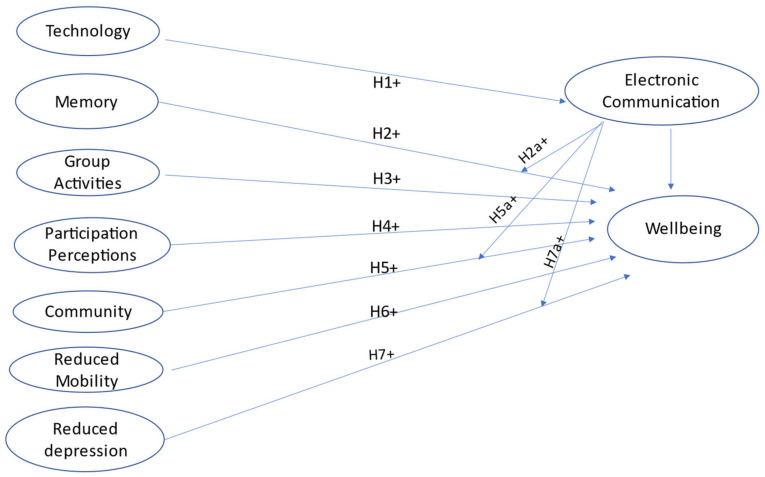
Conceptual Framework on the mechanisms of social support with influencing outcomes on wellbeing. Electronic communication is chosen as the moderating variable moderating the relationship between community, memory, and depression.

The different facets of social support as explained above were examined to determine the correlation with wellbeing. Following the Health people objectives 2030, as in Figure 1, [34] technology or digital health interventions are useful tools to foster group or physical activity and deliver guidance to enhance ADLs [35] and health status of the older adults. The hypotheses are provided below:

**Hypothesis** **H1.** *The use of technology (social network, computer, tablet, and cellphone) is positively associated with wellbeing*.

**Hypothesis** **H2.** *Self-perceived memory is positively associated with wellbeing*.

**Hypothesis** **H2a.** *The use of electronic communication (email and text message) moderates the relationship between cognition as measured by self-perceived memory in NHATS data and wellbeing.*

**Hypothesis** **H3.** *Participatory activities are positively associated with wellbeing*.

**Hypothesis** **H4.** *The perception of participation is positively associated with wellbeing*.

**Hypothesis** **H5.** *Community engagement is positively associated with wellbeing*.

**Hypothesis** **H5a.** *The use of electronic communication (email and text message) moderates the relationship between community engagement and wellbeing*.

**Hypothesis** **H6.** *Help with mobility is positively associated with wellbeing*.

**Hypothesis** **H7.** *Reduction in self-reported depression is positively associated with wellbeing*.

**Hypothesis** **H7a.** *The use of electronic communication (email and text message) moderates the relationship between reduced self-reported depression and wellbeing*.

### 1.5. Theoretical Foundation

This research is informed by the activity theory, drawn from psychosocial aging and gerontology studies. Various digital health interventions can promote group or physical activity among adults 65 years and older and deliver guidance and support that is tailored to individuals’ activity level and health status (Figure 1). The authors considered the theory in the construction of the theoretical model discussed above. The theory supports the maintenance of regular activities and social pursuits as factors contributing to life satisfaction [36]. Older adults make adaptive choices and use strategies tied to past experiences of life [37] and the social world [38] that require connections with family, culture, and social networking [39]. Being socially connected (Figure 1) through technology or community programs promote perceived support, which in turn fosters psychological capital and resilience to stress and physiological impairment [40].

## 2. Methods

### 2.1. Design, Setting, and Sample

This study uses a hierarchical regression analysis on a total of 4623 samples of older adults. We obtained the data from the National Health and Aging Trend Study (NHATS) from year 2018. The software used for analysis was SPSS version 26. The research was approved by the University of North Texas, Institutional Review Board.

The NHATS is a nationally representative sample of 5547 Medicare beneficiaries aged 65 years and older as of 30 September 2014. The data for the current study includes oversamples of black and non-Hispanic people of average age of 79 years and highest age limit of 101 years. Access to the data required registering with the NHATS website and sensitive demographic data, such as age and marital status, were available through a separate application process.

The participant or sample person data set was available in NHATS Round 8 (R8) data files containing responses from the sample person (SP) and the facility questionnaire (FQ) from a total of 6312 observations and 1287 variables. As the key objective of this study was to identify the variable of social support and positive emotions, investigation was conducted with only those contents of the R8 instrumentation questionnaire that included the social support profile of the participants and visualized and reflected upon the frequency and types of relationships in which the participants engaged.

### 2.2. Data Cleaning

The data were cleaned and recoded in SPSS before analysis. All records for independent and dependent variables with responses −1 = inapplicable; −7 = refused to answer; −8 = do not know; and −9 = missing values were removed. A total of 924 samples with more than 10% of null values were deleted, then missing data were imputed by “expectation-maximization” method on the 4623 existing samples for analysis. The variable race was dummy coded. Additional value criteria were added for analysis, and the highest value was given for the highest positive response. For example, for the analysis of the variable of self-perceived memory, the response 5 was recoded to 1 = *poor*; response 4 was recoded to 2 = *fair*; response 3 = *good self-perceived memory* remained the same; response 2 was recoded to 4 = *very good*; and response 1 was recoded to 5 = *excellent*.

### 2.3. Measures

The outcome variable for this study, namely wellbeing, was regressed against the variables of social support as available in the dataset. The predictor variables in Table 1, were investigated to find out whether these indicators were positively associated with wellbeing of the participants. The structure of these indicators matches the proposed indicators of the Healthy People 2030 social domain health objectives based on mental health and healthier social environment The structure of the indicators were replicated from the NHATS data to its entirety.

#### 2.3.1. Wellbeing

The variable of wellbeing is a unidimensional construct in our analysis. As shown in Table 1, the variable wellbeing in the NHATS dataset obtains information about the frequency of feelings of the participants. Questions asked were whether the participant felt confident and good about self. NHATS uses “last month” as the reference period and, in general, fewer response categories. The responses were 1 = every day (7 days a week);2 = most days (5–6 days a week); 3 = some days (2–4 days a week); 4 = rarely (once a week or less); and 5 = never. These responses were recoded towards the positive sentiment as the highest value in this paper. For example, 5 = every day (7 days a week); 4 = most days (5–6 days a week); 3 = some days (2–4 days a week); 2 = rarely (once a week or less); and 1 = never.

#### 2.3.2. Technology

The technological environment in NHATS data refers to the type of communication and information technology available to participants, such as the computer for social networking, use of tablet, or a working cell phone. These items were combined to form a unidimensional factor that was named “technology” in the model. The variable “emailtext”, which includes writing of emails and text messaging in the NHATS dataset, was renamed “electronic communication” in our analysis. Investigation was conducted on whether the use electronic communication moderates the relationship between memory and wellbeing, community and wellbeing, and reduced depression and wellbeing in our model.

In the dataset, questions were asked about the frequency of sending messages by email or texting. The respondents were interviewed with the question, “In the last month, have you ever sent messages by email or texting?” (1 = yes; 2 = no). These responses in the NHATS data had the highest positive value as (yes) 1. The negative value 2 (no) was recoded to 0 in the present study.

#### 2.3.3. Participation

Respondents were interviewed with questions, such as “Besides religious services, did you ever participate in clubs, classes, or other organized activities?” The question rated the positive response as yes = 1 and negative response as no = 2. The negative value 2 (no) was recoded to 0 to facilitate analysis in the present study. The questions on this variable reflect participation in activities that are elective but valued. A unidimensional factor combining various activities as shown in Table 1 was created and named “group activities”. Research [41] confirmed the reliability of the participation measures in the form of a scale; and the reliability of individual items [42] varies in the dataset. This study measured the perceived social support of older adults and named that variable “perception of activities”. Activities with full sequence were measured as very important = 1, somewhat important = 2, or not so important = 3. The full sequence—was the activity done, how important is it to do—was asked for. Questions included “How important it is to do an activity of inclusion?” For example, “How important is it to go out for enjoyment? Would you say very important = 1, somewhat important = 2, or not so important = 3?” In the present study, these responses were recoded as 3 = very important; 2 = somewhat important; and 1 = not so important.

#### 2.3.4. Community

Participants were asked three questions about the community setting: whether people know each other well; whether people are willing to help each other; and whether people in the community can be trusted. The answers were 1 = agree a lot; 2 = agree a little; and 3 = do not agree. Recoding for analysis included changing the responses as 3 = agree a lot; 2 = agree a little; 1 = do not agree. As shown in Table 1, a unidimensional variable for all these three questions was named as “community” in the present model.

#### 2.3.5. Reduced Mobility

The mobility variable includes the key measures of activity limitation in NHATS data [43]. The respondents were asked questions, such as “In the last month, did anyone ever help the participant to go outside of home”? “In the last month, did anyone ever help the participant to get around inside home?” Responses were 1 = yes; 2 = no. The value 2 (no) was recoded to 0 in the present study. As the positive response was given the highest value = 1, mobility was renamed to “reduced mobility” in the model to better reflect it as a social support variable.

#### 2.3.6. Reduced Depression

The variable depression is included in the health conditions section of the NHATS data. The questions that were asked were “Over the last month, how often have you: (a) had little interest or pleasure in doing things; (b) felt down, depressed, or hopeless; (c) felt nervous, anxious, or on edge; (d) been unable to stop or control worrying?” Response categories were 1 = not at all; 2 = several days; 3 = more than half the days; 4 = nearly every day. These responses were recoded as 4 = not at all; 3 = several days; 2 = more than half the days; 1 = nearly every day. In the present study, as the positive response was given the highest value, depression was renamed to “reduced self-reported depression”.

#### 2.3.7. Memory

Memory is included in the cognition section of the dataset and provides information about several aspects of cognitive functioning. Participants were asked to rate their memory currently and the frequency of memory problems related to daily activities. Response categories were 1 = excellent; 2 = very good; 3 = good; 4 = fair; 5 = poor. In the present study, these responses were recoded as 5 = excellent; 4 = very good; 3 = good; 2 = fair; 1 = poor.

### 2.4. Analysis Method

Hierarchical regression determines the amount of variance in the dependent variable (DV) accounted for by a block of variables after accounting for all other variables. The intent was to determine if additional variables significantly improve our model’s ability to predict the criterion variable, “wellbeing”. The analysis conducted investigated the moderating effect of the variable “electronic communication” and its impact on the relationship between community engagement and wellbeing, reduced self-reported depression and wellbeing, and memory and wellbeing of older adults. Hence, hierarchical linear regression analysis was used. The variables were added at separate steps called “blocks” to check the moderating effect and how they predicted wellbeing [44]. The Kolmogorov–Smirnov and Shapiro–Wilks tests investigate the normality in the model. The Durbin–Watson test measures the degree of autocorrelation among the residuals in the regression analysis where such a condition represents a violation of the assumptions required for regression.

## 3. Results

### 3.1. Initial Analyses

An a priori power analysis for linear multiple regression was performed using Gpower 3.1 [45]. The following criteria were chosen for the test parameters because of the directionality of the hypotheses: a one-tailed analysis was selected to detect small differences, a very small effect size of 0.1 was selected, and an alpha of 0.05 and power (1—β) of 0.95 were selected to balance the chance of type I and type II errors. A conceptual model with seven predictors was present in the original analysis. A sample size of 110 was recommended by the analysis, and as the research sample is considerably large, the sample was considered adequate for additional analyses.

### 3.2. Model Reliability and Validity

At the beginning of the statistical analysis, tests for common method bias, reliability, and validity and model evaluation and hypotheses testing were conducted by the researchers. The NHATS data is a national longitudinal survey, and as a result, common method bias is less relevant than traditional single-point-in-time surveys [46]. However, we moved forward with the assessment because we felt it was important to confirm the appropriateness of the data to our application. Harman’s one-factor test was used to assess for the presence of common method bias [47]. An exploratory factor analysis (EFA) was conducted to examine the dimensional structure of all items combined. The analysis revealed that the maximum variance accounted for by a single factor was 14.96%, indicating that common method bias was not present in the data. [48] The Cronbach’s alphas of all the indicators were above 0.07 as shown in Table 2, and they were accepted for hypothesis testing [49].

### 3.3. Model Analysis

Hierarchical regression analysis assumptions were tested. In the scatter plots, the residuals were found to be equal among the regression line; hence, the data passed the test of homoscedasticity and linearity. The Kolmogorov–Smirnov test (0.000) and Shapiro–Wilks test (0.000) were found statistically significant; hence, the error of residuals showed that the data are not normally distributed. As per central-limit theorem assumption, when the sample size is large, as in our model, and the error is not normally distributed, the sampling distribution of the b coefficient will still be normal, so there was no violation of assumptions [50]. The assumption of multicollinearity is examined [51] with the values of variance inflation factor (VIF) to explore the shared variance between independent and dependent variables [52]. Table 3 shows all VIF values are below the acceptable level of 5.0. The observations in these data are independent. The Durbin–Watson statistic is 1.999, which is between 1.5 and 2.5, and therefore, the data are not autocorrelated.

The regression model in Table 3, Table 4 and Table 5 shows that technology, self-perceived memory, group activities, perception of group activities, community engagement, reduced mobility and reduced self-reported depression were statistically significant (*p* = 0.000) predictors of the wellbeing of older adults. The R^2^ is 0.405, and the adjusted R^2^ is 0.403. Hence, the linear relationship of all these variables explains 40.3% of variance on wellbeing. The H1, H2, H3, H4, H5, H6, and H7 are supported in the model.

The interaction effect of electronic communication (emails and text messages) on community engagement (β = 0.027, *t* = 2.018, *p* = 0.044) and reduced self-reported depression are statistically significant (β = 0.032, *t* = 2.667, *p* = 0.008). Hence, it can be said that emails and texting moderates the relationship between community engagement and wellbeing of older adults. Furthermore, it can be said that emails and texting moderates the relationship between reduced self-reported depression and wellbeing of older adults. To this end, H5a and H7a are supported in our analysis. In this model, the relationship between memory and wellbeing are not moderated using electronic communication (β = −0.010, *t* = −1.068, *p* = 0.286). Hence, H2a is not supported.

The variables were selected following the literature as is provided in the NHATS. The data set contains more than 1000 variables. The variables selected in this study were based on the community context as per SDOH following the Heathy People 2030 objectives [35]. These variables are all separate and independent constructs; hence, there is no solid correlation between them. We ran the correlation that worked the best.

The direction of the relationship is also explained in the model. Table 3 explains the coefficients and the *t*-scores of the regression equation. The *t*-scores of the corresponding coefficients evaluate the significance of the independent variables. The first coefficient is constant (intercept) at 1.239. Hence, the expected wellbeing score is 1.239 when everything is held constant. In this model, technology is positively correlated (β = 0.057, *t* = 2.576, *p* = 0.010) with wellbeing, which implies that digital skills do connect the individual to social networks, which in turn contributes to older adult wellbeing. There was a positive relationship found between self-perceived memory (β = 0.049, *t* = 6.892, *p* = 0.000) and wellbeing. Hence, older adults are likely to have more memory power when they are socially included. Group activities (β = 0.184, *t* = 8.535, *p* = 0.000) are positively correlated with wellbeing.

The perception of group activities (β = 0.061, *t* = 6.787, *p* = 0.000) is also positively correlated with wellbeing, which implies that the greater the engagement in activities, the greater the life satisfaction and happiness or wellbeing for older adults. There is a positive relationship between community engagement experience (β = 0.068, *t* = 6.577, *p* = 0.000) and wellbeing; hence, older people living in a community complex that meets the aging care needs, such as that of independent living, assisted living, and skilled nursing care, have a greater chance of wellbeing. Reduced self-reported depression is positively (β = 0.274, *t* = 28.262, *p* = 0.000) associated with wellbeing, which implies that the greater the wellbeing, the fewer depressive symptoms are experienced in older adults. Finally, reduced mobility was negatively (β = −0.013, *t* = −0.707, *p* = 0.000) correlated with wellbeing in this model, which means that less physically active individuals have reduced wellbeing or happiness.

## 4. Discussion

This study showed that the different facets of social support are associated with wellbeing and cognition in older adults, which suggests that group activities and human behaviors are important concerns for happiness and health. Moreover, these activities and perceptions vary with differences in age, marital status, and ethnicity.

As the interviews in the NHATS data were completed in 2015–2016, it is likely that smartphone ownership has become more widespread since then [53], affording older adults’ opportunities to become even more networked individuals. To improve future analytical efficiency, we suggest including questions that directly ask about the redundancy of social networks and precise questions about network measures and media-use patterns. Designing effective interventions to foster social support is difficult since many factors are often beyond the socially isolated senior’s control and are invincible [54]. The existing programs that rely only on robots, volunteers, and donations are unsustainable [55], as any effective intervention requires participant inclusion in the planning, implementation, and evaluation stages for its success [56]. For example, a gatekeeper program for seniors can augment the exchange of social support if the facilitators or coordinators, such as meter readers, utility workers, postal carriers, and librarians, are trained to recognize signs indicating that an older person may need help. Optimum utilization of existing community resources in an age-friendly environment that engages seniors in social activities, such as computer training workshops, luncheons, club activities, farmers markets, etc., also builds community capacity.

Oftentimes, community-based participatory research (CPBR), a collaborative or user-friendly approach that equitably involves all partners in the program and recognizes the unique strengths that each member brings to it, is an effective way to combine knowledge with action [57]. For example, the “Area Agency on Aging” through CPBR can accomplish a change through group activity programs on healthy snacking to improve health outcomes in underserved elderly communities.

Meaningful social connections can be reduced by online social engagement. However, future research on available or affordable resources should keep in mind the boundaries of old age. Recent technological innovations, such as like mHealth, concierge medicine, telemedicine or telehealth, and smart phone applications, such as Snapchat or Uber, are meaningless for older adults without the knowledge and skill for technology use. We also need to know how and to what extent the use of online social media, such as Twitter or Facebook, can be beneficial for older people.

Community activities where the community partners and stakeholders have a cognitive walkthrough to evaluate the system for learnability through exploration [58] can be effective for social support. Technology skill development in an age-friendly environment may open avenues for older adults, mainly for the underserved, to determine their own solutions and meet their needs in their own time [59]. Free weekend technology training workshops, luncheons, club activities in the community centers, senior centers, libraries, or churches can facilitate social connections and foster a useful level of shared experiences of learning and collaboration. Furthermore, the intergenerational service-learning programs may connect students with seniors for social interaction in various ways, such as electronic tutelage or companionship for taking a walk to remain healthy.

Finally, future research may be conducted on how older adults can be counseled on social function changes that may occur at early stages of cognitive loss [60]. The reasons for less participation in community activities may need screening for social engagement to minimize the loss of diverse network ties and a reduce the overarching reliance on narrow interconnected networks, which are typical of old age. Additionally, we need to focus on the clinical side for raising awareness in seniors on the exacerbating effect of perceived social isolation, which often leads to elevated blood pressure and heightened inflammatory or metabolic responses to stress and poor cognition [61]. Socially connected individuals tend to have stronger activation of the ventral striatum of the brain, which shows pleasant pictures of people and fosters happiness.

## 5. Limitations

This study is limited to the responses provided by the samples in the NHATS, so the assessment of an array of social relationships was not possible. We had no information on participants’ life course and social integration prior to study entry. Social relationships and human behaviors are time variant; hence, examination of the long-term effect of social interaction on wellbeing was not investigated. The sample is composed of Medicare beneficiaries and therefore does not represent the uninsured or undocumented, who can be at greater risk for social isolation [62].

As social media constantly evolves with time, new technologies and applications emerge. It is difficult to determine the causal inferences based on the relationships between Internet use and individual behaviors. In addition, it is difficult to define the boundaries of social activities or online network typology. The constructs of technology or wellbeing and perceptions of activity may differ based on participant demographics and technology experiences. The data are based on self-reported responses and hence could include participant bias and dishonesty. Furthermore, the interviewers’ age could have unfavorably biased the older adults’ self-assessments.

## 6. Conclusions

Research on social support to improve wellbeing is sparse. Our findings extend previous knowledge on social interaction and support by demonstrating that group activities, mobility, or the use of technology are associated with wellbeing of older adults. Although the studies are inconsistent on the relationship between group actives and wellbeing, our results showed that social interaction enhance higher cognition, which is a factor to ameliorate wellbeing. In fact, technology skills or social group networking skills gives the senior a greater chance of accessibility to government and health-related services that can reduce individual expenditures as well [63]. Despite the limitations of this study, our findings provide insights for future studies as opportunities of social and community engagement could provide those in need, with a causal pathway to stay organized and maintain the relationship between daily routine and self-management behavior [64]. Assisted-living facilities and nursing homes could organize frequent cultural and group health behavior programs to socially connect seniors. Such steps could also enhance the staff’s cultural competency level and potentially foster the wellbeing of the residents in an age-friendly environment. Finally, the biopsychosocial costs of uncompensated dementia care for the older adult population’s comorbidity explosion cannot be imagined without social communication and the use of online technology.

## Figures and Tables

**Table 1:** Measures of Social Support.

Domain	Indicators of the Social Inclusion (NHATS Interview Items)	Most Positive Response
Community	People know each other well	Yes
	People are willing to help each other	
	People can be trusted	
Depression	Little interest and pleasure	Not at all
	Down, depressed, and hopeless	
	Nervous; anxious	
	Unable to stop worry	
Technology	Working cell phone	Yes
	One phone other than cell	
	Has a working computer	
	Has a tablet computer	
	Online computer use	
Email/Text	Use of email or text messages	Yes
Self-Perceived Memory	Rate your memory	Excellent
	Often, memory problems interfere	
Mobility	Help to go outside	Yes
	Get help inside	
	Family and friends provide help and drive	
	Walk to places	
	Get ride from family and friends	
Group Activities	Ever attend religious services	Yes
	Ever visit friends and family	
	Club meetings and group activities	
	Ever go out for enjoyment	
	Ever go walking	
	Get to do favorite activity last year	
Perception of Group Activities	How important to participate in clubs and groups	Agree a lot
	How important to visit friends and family	
	How important are religious services	
	How important to go out for enjoyment	
Wellbeing	Life has meaning and purpose	Every Day
	Feels confident	
	Often feels full of life	
	Often feels upset	

**Table 2:** Descriptive Statistics and Reliability Test Scores.

Items	Mean	Std. Deviation	Cronbach’s Alpha (α)
Technology	0.62	0.234	0.709
Self-perceived memory	3.61	0.891	0.781
Group activities	0.67	0.232	0.790
Perception group activities	2.19	0.535	0.697
Community engagement	2.42	0.547	0.743
Reduced mobility	0.30	0.219	0.681
Reduced self-reported depression	3.59	0.547	0.743
Wellbeing	2.69	0.340	0.749

**Table 3:** Regression Analysis Predicting Wellbeing of Older Adults: Predictors (N = 4623).

Predictors	Unstd B Coeff	Std β Coeff	*t*-test	Sig	VIF	Hypothesis	Supported
Technology	0.057	0.039	2.576	0.010 *	1.758	H1	Yes
Memory	0.049	0.127	6.892	0.000 **	2.613	H2	Yes
Group activities	0.184	0.125	8.535	0.000 **	1.660	H3	Yes
Perception of group activities	0.061	0.096	6.787	0.000 **	1.535	H4	Yes
Community engagement	0.068	0.109	6.577	0.000 **	2.097	H5	Yes
Reduced mobility	−0.013	−0.009	−0.707	0.000 **	1.143	H6	Yes
Reduced self-reported depression	0.274	0.441	28.262	0.000 **	1.872	H7	Yes

R^2^ 0.405, Adjusted R^2^ 0.403. F for change in Adjusted R^2^ 2.780. * *p* < 0.05. ** *p* < 0.01.

**Table 4:** Regression Analysis Predicting Wellbeing of Older Adults: Moderators.

Moderators	Unstd B Coeff	Std β Coeff	*t*-test	Sig	Hypothesis	Supported
EC x Memory	−0.010	−0.057	−1.068	0.286	H2a	No
EC x Community engagement	0.027	0.099	2.018	0.044	H5a	Yes
EC x Reduced self-reported Depression	0.032	0.167	2.667	0.008	H7a	Yes

**Table 5:** Correlation matrix of study variables.

	DP	CM	TE	CG	MO	GA	PGA	WB
Depression (DP)	1							
Community Engagement (CM))	0.125 **	1						
Technology (TE)	0.208 **	0.130 **	1					
Self-Perceived Memory (CG)	0.317 **	0.096 **	0.227 **	1				
Mobility (MO)	−0.221 **	00.013	−0.185 **	−0.149 **	1			
Group Activities (GA)	0.235 **	0.237 **	0.327 **	0.148 **	−0.079 **	1		
Perception of Group Activities (PGA)	0.134 **	0.221 **	0.198 **	0.117 **	0.011	0.562 **	1	
Wellbeing (WB)	0.564 **	0.201 **	0.246 **	0.306 **	−0.152 **	0.343 **	0.272 **	1

** Correlation is significant at the 0.01 level (2-tailed).

## Data Availability

Our data are readily available upon reasonable request.
